# Case report: Osteomalacia due to bisphosphonate treatment in a patient on hemodialysis

**DOI:** 10.1186/s12882-021-02509-5

**Published:** 2021-09-03

**Authors:** Masaki Hatano, Izuru Kitajima, Seizo Yamamoto, Masaki Nakamura, Kazuya Isawa, Yutaka Hirota, Junichi Hoshino, Naoki Sawa, Yoshifumi Ubara

**Affiliations:** 1 Department of Orthopaedic Surgery, Toranomon Hospital Kajigaya, 1-3-1 Kajigaya, Takatsu, Kawasaki, Tokyo, Kanagawa 212-0015 Japan; 2 Department of Nephrology Center, Toranomon Hospital Kajigaya, 1-3-1, Takatsu, Kawasaki, Tokyo, Kanagawa 212-0015 Japan; 3 Okinaka Memorial Institute for Medical Research, Toranomon Hospital, Tokyo, Japan

**Keywords:** Bisphosphonate, Bone histomorphometry, Etidronate, Hemodialysis, Osteomalacia, Case report

## Abstract

**Background:**

No publications have reported on osteomalacia in patients receiving intermittent cyclical therapy with etidronate (a bisphosphonate) and undergoing long-term hemodialysis (HD).

**Case presentation:**

We report on a 46-year-old Japanese man admitted to our hospital for further examination of left forearm pain. Maintenance HD was started at age 24 years, and the man had been on HD since then. At age 38 years, surgical parathyroidectomy was performed for secondary hyperparathyroidism; iliac crest bone biopsy performed at the same time showed osteitis fibrosa. The active vitamin D_3_ preparation calcitriol was started, and intermittent cyclical etidronate therapy was introduced 2 years later for osteoporosis. At age 45 years, the patient stopped taking calcitriol because of hypercalcemia but continued with etidronate. At age 46 years, a pseudofracture with a Looser zone occurred in the left ulna, and left femur bone biopsy revealed osteomalacia. Etidronate was discontinued, and calcitriol was restarted; open reduction and internal fixation with an angular stability plate were performed. Union of the bone was achieved 10 months after the operation. At age 49 years, a lumber bone biopsy confirmed improved bone morphometry.

**Conclusions:**

We believe that intermittent cyclical etidronate therapy without administration of active vitamin D_3_ during long-term HD might have induced osteomalacia, resulting in the ulna insufficiency fracture. Therefore, we propose that administration of active vitamin D_3_ is essential to prevent osteomalacia in patients on long-term HD who are receiving bisphosphonates and have potential vitamin D_3_ deficiency.

## Background

Mineral abnormalities related to renal osteodystrophy (ROD) are common in patients with chronic kidney disease (CKD) [1]. The gold standard for the diagnosis and specific classification of ROD is bone biopsy with bone histomorphometry [2]. A bone biopsy is recommended if results will guide treatment decisions, and is therefore advised in cases of unexplained fractures and bone pain, and may be considered prior to PTX or starting anti-resorptive treatment. However, a bone biopsy is not well suited to monitor treatment response in cases of frequent changes, as bone is a slow-reacting tissue.

Bisphosphonates (BPs) are bone antiresorptive agents that are widely used to treat postmenopausal or glucocorticoid-induced osteoporosis. The current Kidney Disease: Improving Global Outcomes (KDIGO) recommendations limit their use in patients with renal impairment because of concerns about worsening renal function, bone mineralization defects, and osteomalacia. However, the pathogenesis of osteomalacia due to BPs in HD patients remains unclear. Currently, no publications report on osteomalacia due to BPs in patients on long-term HD. Here, we describe a patient on long-term HD who received intermittent cyclical etidronate therapy after parathyroidectomy (PTX) and developed osteomalacia after discontinuing active vitamin D_3_ derivative.

## Case presentation

**Fig. 1:**
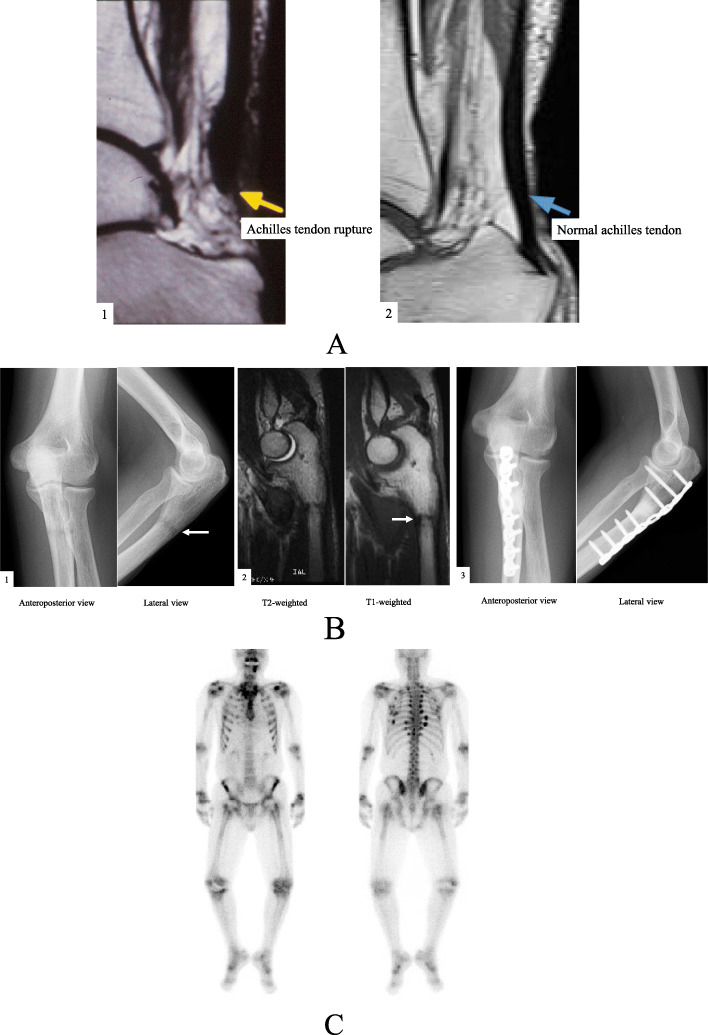
(**A**) 1 Sagittal t1 magnetic resonance imaging (MRI) shows complete Achilles tendon tear (yellow arrow); 2 Sagittal t1 MRI shows Normal achilles tendon (blue arrow). (**B**) 1 Anteroposterior and lateral radiograph of the left ulna shows left ulna pseudofracture (white arrows); 2 Sagittal T1 and T2 weighted images in MRI show low band in the bone; 3 Successful bone union. (**C**) Bone scintigraphy revealed multifocal lesions, including multiple ribs, costochondral junctions, costovertebral junctions, and both posterior iliac bones

**Fig. 2:**
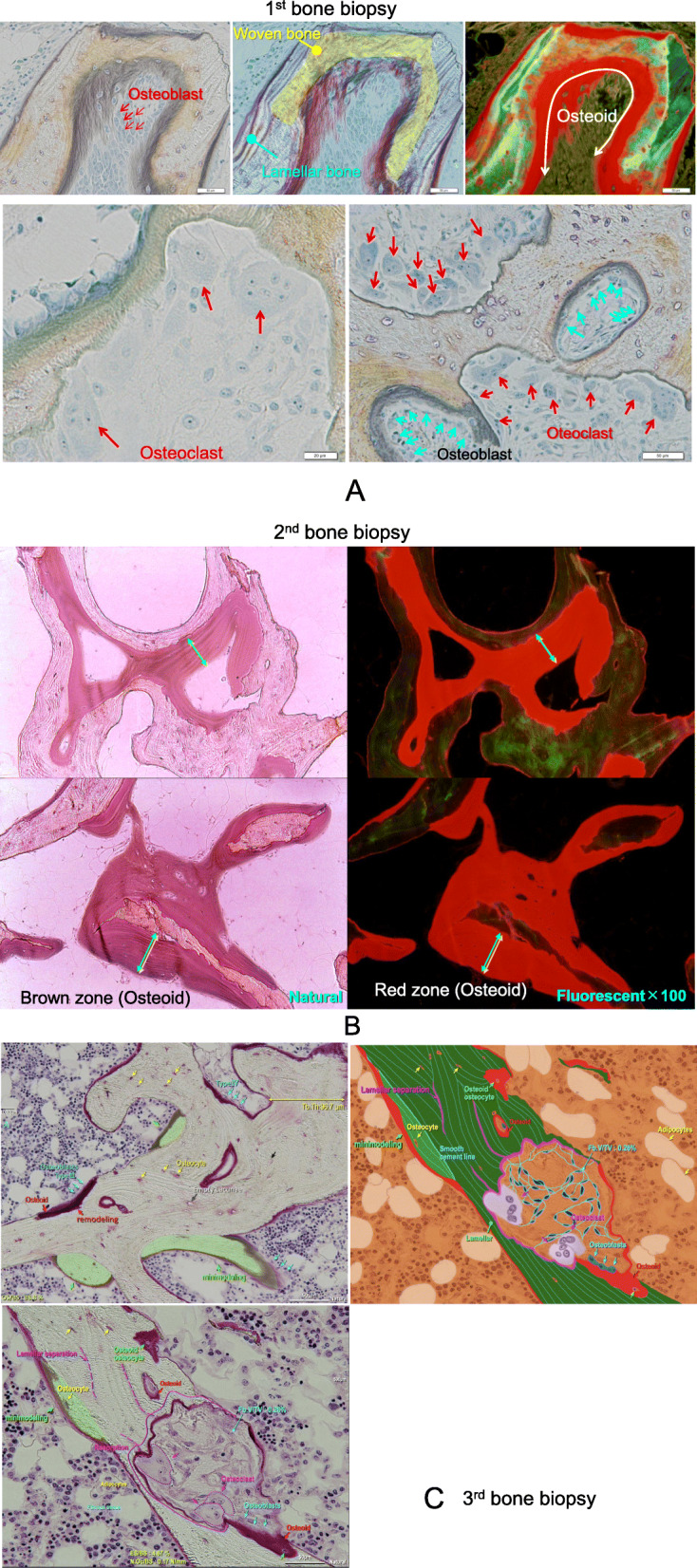
(**A**) Natural light and fluorescent microscopic analysis of the iliac bone section (1st bone biopsy). Bone resorption by osteoclasts and bone formation by osteoblasts progress at the same time, and woven bone was formed characterized by haphazardly organized collagen fibers. (**B**) Natural light and fluorescent microscopic analysis of the femoral cortical bone near a cancellous bone section (2nd bone biopsy). An increase in the amount of osteoid was observed, along with increased thickness of osteoid seam width and a low calcification area on the bone surface and in the bone. (**C**) Analysis of the lumbar spine bone section (3rd bone biopsy). Both remodeling and minimodeling were observed. The majority of trabecular bone was occupied by 2 types of new lamellar bone formation: remodeling, which is characterized by new bone formation by osteoblasts after bone resorption by osteoclasts, and minimodeling, which is characterized by a lack of precedent bone erosion by osteoclasts

A 46-year-old Japanese man on HD for 22 years was admitted to our hospital for further examination of left forearm pain. Maintenance dialysis was started at age 24 years after bilateral nephrectomy for hemorrhagic angiomyolipoma related to tuberous sclerosis. At age 38 years, the patient developed severe bone pain without any precipitating cause, and bilateral Achilles tendon rupture was noted (Fig. 1A). Because secondary hyperparathyroidism (with an intact parathyroid hormone [PTH] level of 1630 pg/mL) resistant to conservative treatment was considered a causative factor, surgical PTX was performed with autotransplantation into the subcutaneous tissue of the non-shunt side forearm. The iliac crest bone was biopsied at the same time and bone histomorphometric analysis was performed at the Ito Bone Science Institute, Niigata, Japan. Tetracycline double labeling was also performed with doxycycline at 200 mg daily (with a schedule of 3 days on, 12 days off, 3 days on, 13 days off). Osteitis fibrosa was diagnosed, with a fibrous tissue volume to total volume (Fb.V/TV) of 9.1% (> 0.5% required for diagnosis) and an osteoid volume to bone volume (OV/BV) of 13.0% (< 15% required for diagnosis according to the Sherrard et al. classification of ROD) [3]. Double labelling by tetracycline showed a bone formation rate per unit of bone volume (BFR/BV) of 120.2% per year, indicating a high bone turnover. Bone is resorped by osteoclasts and formed by osteoblasts, and normally these 2 processes progress at the same time and rate; in our patient, the higher bone turnover resulted in woven bone characterized by haphazardly organized collagen fibers (Fig. 2A).

After PTX, the intact PTH level was less than 100 pg/mL, so the patient was prescribed calcium lactate (3.0 g/d) and active vitamin D_3_ (calcitriol, 0.5 μg/d) to treat hungry bone syndrome. The bilateral Achilles tendon rupture healed. Two years after the surgical procedure, low bone mineral density was diagnosed (T-score of − 3.1 measured by dual energy X-ray absorptiometry [DEXA]) and intermittent cyclical etidronate therapy (200 mg/d for 2 weeks every 4 months) was started; calcitriol was continued. At age 45 years, calcitriol was stopped because of hypercalcemia, and at age 46 years the patient developed left forearm pain for no obvious reason that worsened by the day. One week later, a radiograph showed a pseudofracture with a Looser zone in a proximal portion of left ulna; the pseudofracture manifested as a 3 mm-wide radiolucent band perpendicular to the bone surface (Fig. 1B). Therefore, the patient was admitted to our hospital for surgical treatment.

On admission, the patient was 174.0 cm tall and weighed 79.5 kg. Blood levels (Table 1) were as follows: the patient had PTH in target range, with normal calcium, high phosphate levels and lower 1.25-dihydroxy vitamin D_3._

**Table 1:**
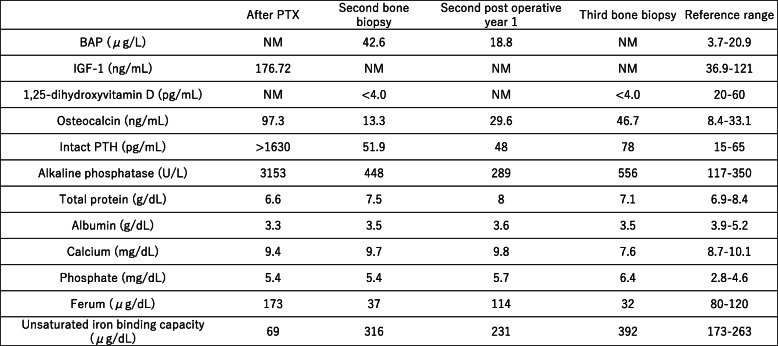
Laboratory Data. BAP: bone alkaline phosphatase, IGF-1: Insulin-like growth factor 1, NM: no measurement, PTH: parathyroid hormone, PTX: parathyroidectomy

Bone scintigraphy with ^99m^Tc-labeled methylene diphosphonate showed intense uptake in multifocal regions, including multiple ribs, costochondral junctions, costovertebral junctions, and both posterior iliac bones, as well as in the affected left ulna; these findings are characteristic of osteomalacia (Fig. 1C).

Open reduction and fixation with an angular stability plate and left total knee arthroplasty were performed. At the same time, left femur bone biopsy was performed to identify the pathogenesis of the ulna pseudofracture. Bone histomorphometric analysis was performed at the Ito Bone Science Institute (Niigata, Japan). Tetracycline double labeling was not performed.

Cancellous bone was assessed by bone histomorphometry (Table 2). The trabecular bone volume (BV/TV) was within the age-matched reference range according to the report by Reccker et al. [4]; however, the trabecular thickness (Tb.Th) was lower than at the first bone biopsy (93.4 μm). All osteoid markers were increased compared with the first bone biopsy, parameters of bone resorption were decreased (Fig. 2B). Osteomalacia was diagnosed according to Sherrard’s classification of ROD [3] because the Fb.V/TV was 0.1% (< 0.5% required for diagnosis) and the OV/BV was 40.7% (> 15% required for diagnosis). The number of osteoclasts (N.Oc/BS) was decreased to 0.148 N/mm, and the number osteoblasts surface (Ob.S/BS) was 12.3%.

**Table 2:**
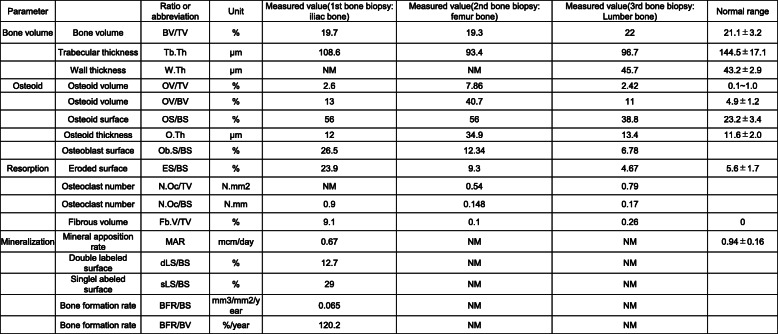
First, second, and third histomorphometric analysis of the iliac crest

A deficiency in 1,25-dihydroxyvitamin D was found, and we assumed that this deficiency, combined with administration of etidronate in a condition of lower PTH levels after PTX, might have contributed to mineralization disturbance-related osteomalacia and resulted in the formation of multiple pseudofractures, including a Looser zone because bone disease manifested 1 year after calcitriol was stopped. Therefore, calcitriol was restarted at a dose of 0.5 μg/d and etidronate was discontinued. Ten months later, the Looser zone had disappeared and bone union was achieved (Fig. 1B).

At age 49 years, a surgical procedure was performed for lumbar destructive spondyloarthropathy related to beta 2 microglobulin amyloidosis resulting from long-term HD, and a lumber bone biopsy was performed at the same time. Bone histomorphometric analysis was again performed at the Ito Bone Science Institute (Niigata, Japan). Tetracycline double labeling was not performed.

At the third bone histomorphometric analysis, all osteoid markers had improved compared with the second biopsy. The abnormal woven bone seen at the first biopsy and abnormal osteoid seen at the second biopsy had disappeared. Normal levels of osteoblasts and osteoclasts were noted. The majority of trabecular bone was occupied by lamellar bone, which is obtained by well-balanced bone remodeling and indicates new bone formation by osteoblasts arising after bone resorption by osteoclasts. In addition, new bone had been formed by minimodeling, which is characterized by a lack of precedent bone erosion by osteoclasts (Fig. 2C) [5].

Bone mineral density (BMD) assessed by DEXA at the lumbar spine and the forearm were improved (Table 3).

**Table 3:**
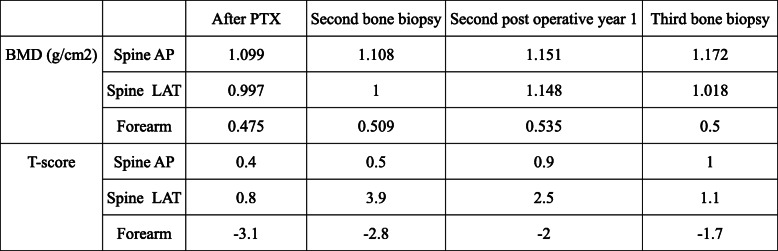
Bone mineral density data. PTX: parathyroidectomy, Spine AP: Spine anterior-posterior, Spine LAT: Spine lateral

## Discussion and conclusions

These findings indicated that active vitamin D_3_ replacement induced normal bone formation even though PTH levels were lower after PTX [6].

Chronic kidney disease mineral and bone disorder (CKD-MBD) is a complex disorder of bone and mineral metabolism. Stress fractures are known to be caused by ROD associated with bone fragility and long-term HD [7, 8]. Therefore, ROD must be evaluated and diagnosed: According to histologic features of biopsied bone, ROD is classified as osteitis fibrosa, osteomalacia, and mixed, mild, and adynamic disease [3]. Generally, BMD is widely used to evaluate osteoporosis; however, it does not predict the type of ROD or accurately evaluate bone fragility in HD patients [9]. KDIGO recommends that bone biopsy should be considered if it will inform treatment decisions [10]. The decision to treat with either antiresorptive or anabolic agents needs to be based on the level of kidney function, bone turnover, and mineralization.

BPs are potent inhibitors of osteoclast-mediated bone resorption. They are widely used to treat postmenopausal or glucocorticoid-induced osteoporosis, but little evidence is available on their effectiveness and safety in patients on HD. Previous studies reported that BPs were a potential treatment for renal osteodystrophy, particularly in patients with high bone turnover or hypercalcemia related to increased release of calcium from bone [11, 12], and that they may be beneficial in HD patients [13, 14].

BPs are classified into 2 groups with different molecular modes of action: non–nitrogen-containing BPs and nitrogen-containing BPs. Etidronate is a non–nitrogen-containing BP. BPs have several severe adverse effects, including acute renal failure, worsening renal function, reduced bone mineralization, and osteomalacia. The pathophysiological mechanisms by which BPs cause osteomalacia are still unknown. So far, studies have reported that etidronate and clodronate, another non–nitrogen-containing BP, may inhibit bone mineralization [15–17]. Reports on osteomalacia or mineralization defects associated with etidronate therapy were described in patients with Paget’s disease who received continuous etidronate therapy at high doses (17–20 mg/kg/d). High doses and continuous therapy with etidronate might induce mineralization defects, although no serious adverse effects were reported with intermittent cyclical etidronate therapy for postmenopausal osteoporosis [18].

Osteomalacia is a bone disease characterized by low bone turnover, defective mineralization, and accumulation of unmineralized osteoid. Traditionally, ROD-related osteomalacia was caused by aluminum toxicity (aluminum inhibits bone mineralization in case of vitamin D deficiency) [3], and osteomalacia caused by iron deposition was frequently found from 1970 to 1990. Hypocalcemia, hypophosphatemia, hypovitaminosis D, toxic effects of drugs are common in patients with CKD [19, 20]; however, few studies reported finding osteomalacia due to vitamin D deficiency in HD patients [8, 21, 22]. In addition, the effects of vitamin D deficiency in HD patients remain to be clarified.

Our patient started intermittent cyclical etidronate therapy after surgical PTX for severe secondary hyperparathyroidism. After PTX, our patient took the active vitamin D_3_ preparation calcitriol because of hungry bone syndrome [23]. However, 7 years after PTX calcitriol was discontinued because of hypercalcemia. One year later, the ulna pseudofracture occurred and osteomalacia was diagnosed. In this case, intermittent cyclical etidronate therapy combined with vitamin D deficiency and HD might have contributed to bone demineralization.

Secondary hyperparathyroidism is a common problem in long-term HD patients. Despite considerable advances in medical therapy for secondary hyperparathyroidism, PTX remains an important therapeutic tool for treating refractory hyperparathyroidism. Surgical PTX is better at preventing recurrence than other surgical therapies [24]. On the other hand, PTX can easily induce hypocalcemia and adynamic bone disease [25, 26], and even now no appropriate pharmacotherapy exists for low PTH levels after surgical PTX. Minimodeling was reported to be closely related to new bone formation in patients with hypoparathyroidism [5].

This study has limitation. Thus patient’s underlying tuberous scleros may be contributed to the bone abnormalities, because tuberous sclerosis can cause bone cysts or sclerotic lesions.

In conclusion, the first biopsy in our patient showed that osteitis fibrosa related to hyperparathroidism contributed to the Achilles tendon ruptures. The second biopsy showed that osteomalacia contributed to the pseudofracture of the ulna, seen as a Looser zone; the osteomalacia was related to continuation of etidronate and 1,25-dihydroxyvitamin D deficiency resulting from discontinuation of calcitriol in a condition of low serum levels of PTH after PTX. The third biopsy showed that resumption of calcitriol and discontinuation of etidronate improved the bone disease, despite continued low levels of PTH. This case indicates that when BPs are started in long-term HD patients with a potential vitamin D deficiency administration of active vitamin D_3_ is essential to prevent osteomalacia via mineralization disturbances.

## Data Availability

All data generated or analysed during this study are included in this published article.

## Abbreviations

ALP: Alkaline phosphatase
BAP: bone alkaline phosphatase
BFR/BV: bone formation rate per unit of bone volume
BMD: bone mineral density
BPs: bisphosphonates
BV/TV: trabecular bone volume to total volume
CKD: chronic kidney disease
CKD-MBD: chronic kidney disease mineral and bone disorder
DEXA: dual energy X-ray absorptiometry
ES/BS: eroded surface to bone surface
Fb.V/TV: fibrous tissue volume to total volume
HD: hemodialysis
KDIGO: Kidney Disease: Improving Global Outcomes
N.Oc/BS: number of osteoclasts to bone surface
Ob.S/BS: osteoblasts surface to bone surface
OS/BS: osteoid surface to bone surface
O.Th: osteoid thickness
OV/BV: osteoid volume to bone volume
OV/TV: osteoid volume to tissue volume
PTH: parathyroid hormone
PTX: parathyroidectomy
ROD: renal osteodystrophy
Tb.Th: trabecular thickness
Th.W: trabecular unit wall thickness
